# Comment on ‘Acute Sarcopenia: Systematic Review and Meta‐Analysis on Its Incidence and Muscle Parameter Shifts During Hospitalisation’ by Aldrich et al.: The Authors' Reply

**DOI:** 10.1002/jcsm.13863

**Published:** 2025-06-04

**Authors:** Luke Aldrich, Theocharis Ispoglou, Konstantinos Prokopidis, Jasem Alqallaf, Oliver Wilson, Antonis Stavropoulos‐Kalinoglou

**Affiliations:** ^1^ Carnegie School of Sport Leeds Beckett University, Headingley Campus Leeds UK; ^2^ Institute of Life Course and Medical Sciences University of Liverpool Liverpool UK

We appreciate the opportunity to respond to the comments raised by Silva and Cipriano [1] regarding our recently published systematic review and meta‐analysis on acute sarcopenia [2]. Constructive discussions such as this help clarify methodological considerations and enhance scientific understanding.

Firstly, regarding knee extensor strength measurement, Silva and Cipriano [1] note that our review stated their study [3] did not assess knee extensor strength. We acknowledge that they measured strength using neuromuscular electrical stimulation to evoke peak force. However, our review excluded studies using electrically evoked contractions, as voluntary contractions are the recognised standard for sarcopenia assessments. This exclusion criterion was applied consistently across all studies, ensuring methodological alignment. Although we did not explicitly state this in our inclusion/exclusion criteria, we appreciate the opportunity to clarify this point. To further justify our stance on excluding this, we would like to highlight conclusions from Jenkins et al. [4] who acknowledged that voluntary and evoked contractions offer unique information to each other and should not be used interchangeably.

Secondly, with reference to the sample size inclusion, we included only the control group from Silva et al.'s study, as per our inclusion criteria that specified that only control groups from intervention studies would be included. Although their total sample size was 60 participants, we reported only the control group (*n* = 30) as our review focused on muscle changes during hospitalisation independent of interventions. We acknowledge that this distinction could have been stated more clearly.

We respectfully disagree with the claim that our review contains inaccuracies that could compromise the integrity of the scientific record. Our methodological decisions were carefully considered, transparent, and aligned with standard sarcopenia assessment practices. The exclusion of electrically evoked contraction measures was a deliberate methodological choice, as voluntary contractions are the established standard. Although this criterion was not explicitly outlined in our inclusion/exclusion criteria, it reflects a methodological decision rather than an inaccuracy. We appreciate the opportunity to clarify this and believe our approach remains clear to researchers familiar with standard assessment methods.

We thank Silva et al. for their engagement and the opportunity to address these points.

## Ethics Statement

The authors have nothing to report.

## Conflicts of Interest

The authors declare no conflicts of interest.

## Data Availability

The authors have nothing to report.
